
JOURNAL INFORMATION
==============================
NLM Title Abbreviation: Buffalo Med J
NLM Journal ID: 0027534

Buffalo Medical Journal

ISSN: 1040-3817
Publisher: Biomedical Journal Digitization Project

ARTICLE INFORMATION
==============================
PMCID: PMC8768149
PMID: 36888230
Article version: 1
Subjects: Miscellany

Miscellany

Print publication date: 1904 May
Volume: 43
Volume Series: New Ser
Issue: 10
First page: 716
Last page: 716
License: This work is free of known copyright restrictions. For more information, please see PMC Back Issue Digitization.
License URL: https://creativecommons.org/publicdomain/mark/1.0/

==============================

Annual Report of the Surgeon-General of the Public Health and Marine Hospital Service of the United States. For the fiscal year 1902. Washington: Government Printing Office. 1903.

Medical Union Number Six. By William Harvey King. Sextodecimo, 60 pages. The Monograph Press, New York. 1904. (Price, boards, 35.)

---

### MISCELLANY.

"The Declaration of Independence," in colors with facsimile signatures, is being sent out by the Palisade Manufacturing Company, Yonkers. It is the design to reach every physician in the United States. We are requested to announce that if any are missed or should fail to receive a copy, the error will be corrected, if the company is notified. It is a souvenir worth possessing.

---

P. Blakiston's Son & Company announce that in printing all the copies of *Deaver's Surgical Anatomy*, so far demanded by its most successful sale, there will have been used 2,340 pounds of ink, 188,002 pounds or 84 tons of paper, and the printing press will have made 3,455,000 impressions. On and after July 1, 1904, the price of this work will be advanced to $30 in half morocco, and $33 in half Russia binding.

---

The Maltine Company, of Brooklyn, has issued for several years past one of the most convenient and substantial visiting lists for physicians. The quality of the paper and its weight, its flexible binding in black seal leather, and its ornamentation in gilt all combine to make it a most practicable clinical memorandum book for the busy doctor. Application to the Maltine Company, mentioning this journal, and enclosure of return postage, will secure a copy to any physician.

---

The Perpetual Visiting and Pocket Reference Book is the title of a convenient memorandum book for a physician, prepared and published by the Dios Chemical Company, Saint Louis. It contains the usual emergency reminders and clinical data required in daily practice and a weekly call list, the dates of which can be inserted by the physician. It is bound in black leather and will be sent free by the publisher on receipt of 10 cents to pay postage.

---

FOR SALE.—A good general practice at Niagara Falls, N. Y., established 17 years. With or without residence, office furniture, hour sign, etc. For particulars, apply to A. G. Sage, M. D., 12 Parker avenue, Buffalo, N. Y.
